# Increased circulating heat shock protein Hsp70 serum levels as a potential biomarker in bronchial asthma patients

**DOI:** 10.1038/s41598-025-28297-8

**Published:** 2025-11-18

**Authors:** Ella Churyukina, Elena Oganesyan, Olga Ukhanova, Inga Kotieva, Marina Gulyan, Elena Koreeva, Ekaterina Portnyaga, Danila Bobkov, Stephanie E. Combs, Maxim Shevtsov

**Affiliations:** 1 Federal Rostov State Medical University of the Ministry of Health of the Russian Federation, 29, Nakhichevansky Lane, Rostov-on-Don, 344022 Russia; 2 Federal Kuban State Medical University of the Ministry of Health of the Russian Federation, 4, M. Sedina str, Krasnodar, 350063 Russia; 3 Institute of Cytology of the Russian Academy of Sciences (RAS), Saint Petersburg, 194064 Russia; 4 Federal Stavropol State Medical University of the Ministry of Health of the Russian Federation, 310 Mira str, Stavropol, 355017 Russia; 5 Federal North Caucasus Federal Scientific and Clinical Center, FMBA of Russia, 24 Sovetskaya str, Yessentuki, 357600 Russia; 6 State Autonomous Institution of the Rostov region «Regional Consultative and Diagnostic Center», Rostov-on-Don; 127 Pushkinskaya str, Rostov-on-Don, 344000 Russia; 7 SM-Clinic, 13, 2nd Zvenigorodskaya str., p. 40, Moscow, 123022 Russia; 8 Department of Immunobiology and Biomedicine, Scientific Center of Genetics and Life Sciences, Federal Territory Sirius, Sirius University of Science and Technology, Krasnodar Krai, 354340 Russia; 9 Department of Radiation Oncology, Technische Universität München (TUM), Klinikum rechts der Isar, 81675 Munich, Germany

**Keywords:** Heat shock protein 70, IgE, ILs, TSLP, Bronchial asthma, Diagnostic marker, Biomarkers, Diseases, Immunology, Medical research

## Abstract

The 70 kDa major stress-inducible member of the heat shock protein 70 family Hsp70 plays an important pathogenic role in inflammatory and autoimmune diseases, including bronchial asthma, and therefore the detection of the levels of the circulating chaperone in serum or plasma could be employed as a diagnostic and prognostic marker. The prospective open single-center study enrolled 78 adult bronchial asthma patients and age-matched healthy volunteers (*n* = 78). Serum HSP70 levels were measured using the ELISA Kit. Serum concentrations of HSP70 as detected by the R&D Systems Hsp70 ELISA in asthmatics patients were significantly higher as compared to control subjects constituting 31.2 ng/ml (*p* < 0.001) and were negatively correlated with FEV1 (forced expiratory volume in 1 s) and FEV1/FVC in all patients with bronchial asthma. When compared the chaperone levels in non-eosinophilic and eosinophilic (> 150 cells/µL) asthma patients, statistical significance was detected (*p* < 0.05). Analysis revealed a significant increase of cytokines (IL-4, IL-17, IL-25, IL-33) and TSLP in bronchial asthma patients. Strong correlation was observed between the Hsp70 level and smoking (*p* < 0.001), but not with age and gender: In conclusion, increased Hsp70 levels in serum of asthmatic patients may serve as a potential biomarker of the disease pending further validation.

**Supplementary Information:**

The online version contains supplementary material available at 10.1038/s41598-025-28297-8.

## Introduction

The pathogenesis of immune-mediated diseases, including various phenotypes of bronchial asthma, is particularly determined by an aberrant response of the body’s immune system involving various effector cells and molecules^1^. In turn, identification of specific participants in the pathogenesis of the disease and their role in its development will allow us to identify both diagnostic/prognostic markers and promising targets for therapy. Molecular chaperones, especially representatives of the broad-studied HSP70 family, in addition to participating in the regulation of cellular proteostasis (assembly and disaggregation of polypeptides and proteins), participation in apoptosis and cellular autophagy, also play an important immunological role, acting as DAMP molecules^2–4^. It has been previously shown that extracellular HSP70 (secreted by cells as part of extracellular vesicles or in dissolved form) is able to activate both innate and acquired immune responses^5–7^. Molecules such as TLR2, TLR4, CD40, LRP/CD91, c-type lectin family member (DC-SIGN) closely related to LOX-1 have been identified as potential HSP70 receptors^8^. Thus, the interaction of the chaperone with CD14 on human monocytes upregulated the expression of pro-inflammatory cytokines such as tumor necrosis factor (TNF)-alpha, interleukin (IL)-1beta and IL-6^9^.

Given the increased production of HSP70, this protein has been actively studied as a potential diagnostic and prognostic biomarker for various diseases including cancer, arterial hypertension, diabetes, infectious and autoimmune diseases, etc^10–16^. Apart, from these studies elevated levels of Hsp70 (or plasma antibodies recognizing chaperone) were also previously reported in bronchial asthma patients^17,18^. In vitro and in vivo studies have clearly demonstrated the involvement of HSPs in the pathogenesis of airway inflammation in asthma, which affects normal cells residing in the bronchial airways (e.g., bronchial epithelial cells, alveolar macrophages) as well as cells of the adaptive and innate immune system^19^. As shown by Fang et al., the intracellular interaction of Hsp70 with myristoylated alanine-rich C kinase substrate (MARCKS), a key regulator controlling mucin secretion by airway epithelial cells, mediates mucin secretion in an in vitro model using normal human bronchial epithelial cells (NHBE)^20^. Elevated levels of extracellular Hsp70 were detected in exosomes isolated from bronchoalveolar lavage fluid (BALF) of mice that developed tolerance to allergens. Moreover, it was shown that prophylactic intranasal immunization of mice with such exosomes prevented the development of allergic airway inflammation induced by sensitization and allergen challenge with olive pollen^21^. On the other hand, it has been shown in vitro that Hsp70 can stimulate neutrophil chemotaxis^22^. In addition, one of the characteristics of HSP70 as a “danger signal” is the ability to activate neutrophils through association with TLR4^23^. In the study by Tamási et al. the authors reported the elevated Hsp70 serum levels in asthmatic woman (*n* = 40) during the gestation period as compared to healthy pregnant woman, constituting (median (25–75 percentile) 0.44 ng/ml (0.36–0.53) versus 0.21 ng/ml (0–0.27), *p* < 0.001), respectively^24^. Further studies showed that serum Hsp70 that do reflect the oxidative stress and inflammation and are elevated in patients with preeclampsia, particularly in patients with HELLP syndrome (hemolysis, elevated liver enzymes, low platelet count)^25–27^. Indeed, when Hsp70 levels were analyzed in asthmatic patients elevated concentrations of chaperone were significantly increased in plasma, 0.46 ng/ml versus 0.14 ng/ml (control) and in sputum, constituting 0.88 ng/ml versus 0.42 ng/ml (control)^28^. In a more recent study by Huang et al. the authors reported increased levels of several DAMP molecules including Hsp70 (as well as HMGB1, LL-37, and S100A8) in sputum of asthma-COPD overlapped patients^29^.

In the current study the level of Hsp70 was assessed in correlation with the cytokines (i.e., IL-4, IL-17, IL-25, IL-33, TSLP) which are known to play a major role in the pathogenesis of asthma^30–33^. According to the recently proposed “epithelial barrier hypothesis”, the damage by various environmental factors (aeroallergens, pathogens, pollutants) of epithelial barriers, in particular, the respiratory tract, leads to the secretion of cytokines – alarmins – by epithelial cells. These cytokines induce initiation of various inflammatory cascades, ultimately forming the heterogeneous nature of inflammation in asthma^34^. Among alarmins, the following cytokines are of special importance including thymic stromal lymphopoietin (TSLP), interleukins IL-4, IL-17, IL-25 and IL-33, which determine a special variant of activation of antigen-presenting dendritic cells (DC), directing the differentiation of T-helper lymphocytes (Th), activated by an allergen, along the proallergic Th2 and Th9 pathways. Additionally, these cytokines cause the activation of lymphoid cells of innate immunity type 2 (ILC2), which resemble Th2 in the spectrum of synthesized cytokines, forming in both cases Th2 (eosinophilic) inflammation^35,36^. Furthermore, these molecules might trigger either non-allergic Th17 and Th22 signaling pathways, with the development of “potentially neutrophilic” (non-Th2) inflammation, or they launch a cascade, multifacetedly affecting fibroblasts, which ends with the formation of “T2 and non-T2 inflammation”^37^. Early detection of cytokines in patients with bronchial asthma as well as identification of new diagnostic/prognostic biomarkers of the inflammatory cascade mirrowing the early stages of T2 and non-T2 inflammation, could be employed for the development of therapies which in turn will help to prevent the irreversible remodeling of the airways^37^.

The initial hypothesis suggests, taking into account the immunomodulating functions of the protein and its role as a molecule of alarmine, the increased content of chaperone in patients with bronchial asthma. In the present prospective, non-blinded, single-center study the serum chaperone content was analyzed by the R&D Systems Hsp70 ELISA (R&D Systems, USA) in adult patients with bronchial asthma and an increased protein content was demonstrated in this group of patients. Furthermore, we performed correlation analysis of the elevated levels of Hsp70 with other markers associated with BA pathogenesis (i.e., IL-4, IL-17, IL-25, IL-33, TSLP).

## Results

### Patients characteristics

Clinical characteristics of the asthmatic patients are presented in Table 1. The age of the patients ranged from 18 to 65 years. The average age of the entire cohort did not differ between the asthma group and the control group and was 45 years (45.0 ± 1.5 years, for women 43 years, for men 46 years versus 44.92 ± 2.9, for women 42 years, for men 45 years). Among the patients, the number of females was 47.7% (*n* = 37, males 52.3% (*n* = 41)). The duration of the disease varied from 3 to 20 years or more. The average duration of the disease constituted 10.1 ± 0.7 years.

A heredity burdened by allergopathology was present in 42% (33 people) of the studied patients. In 58% (45 people), heredity was not burdened by allergopathology. 60.8% patients had concomitant allergic diseases in the past or in the present.

**Table 1:** Asthmatic patients and subject demographics.

Characteristic	Bronchial asthma (BA) patients	Control group
Number (n)	78	78
Gender		
Male (n/%)	41 / 53%	40 / 51%
Female (n/%)	37 / 47%	38 / 49%
Age (years)	45 (18–65)	46 (18–65)
Smoking history		
Never	30	40
Current	10	8
Ex-smokers	10	10
BMI (body mass index)		
Normal weight 18.5–24.9 (n / %)	0	68 / 87%
Overweight 25.0–29.9 (n / %)	2 / 2%	8 / 10%
Obesity class I 30.0–34.9 (n / %)	5 / 7%	1 / 1.5%
Obesity class II 35.0–39.9 (n / %)	15 / 20%	1 / 1.5%
Obesity class III > 40 (n / %)	53 / 71%	0
Eosinophilic asthma (n /%)	43 / 61%	N/A
Non-eosinophilic asthma (n / %)	28 / 39%	N/A
Types of BA asthma		
J45.0 Allergic (J45.0)	18 / 32%	0
J45.8 Mixed (J45.8)	34 / 61%	0
J45.9 Non-identified (J45.9)	4 / 7%	0
Peripheral blood counts		
Lymphocytes, % / cell/µl	49%/4031.8	19,81% /1484.55
Neutrophils, % / cell/µl	43%/ 3493.4	53.78% / 2455.24
Eosinophils, % / cell/µl	7.5% / 613.1	3.83% / 89.65
Basophils, % / cell/µl	0.5%/27.8	1.38% / 32.13
Concomitant diseases		
AERD# (n /%)	12 / 41%	0
Atopic dermatitis (n /%)	8 / 28%	0
Allergic rhinitis (n /%)	9 / 31%	0
Therapies		
SABA^##^ (n /%)	20 / 25.6%	0
Low dose ICS (≤ 250 µg fluticasone equivalent)* (n /%)	24 / 30.76%	0
ICS, moderate dose ** (n /%)	28 / 35.9%	0
High dose ICS (> 500 µg fluticasone equivalent)*** (n /%)	26 /33.3	0
LABA# (n /%)	58 / 74.4%	0
Tiotropium bromide (n /%)	22 / 28.2%	0

^#^*AERD* -- aspirin-exacerbated respiratory disease.

*Low dose ICS (≤ 250 µg fluticasone equivalent).

**ICS, moderate dose (n /%).

***high dose ICS (> 500 µg fluticasone equivalent).

ALR – leukotriene receptor antagonists.

^##^*SABA* short-acting β2 agonist.

^##^*LABA* long-acting β2 agonist.

### Circulating serum HSP70 is increased in asthmatic patients

**Fig. 1:**
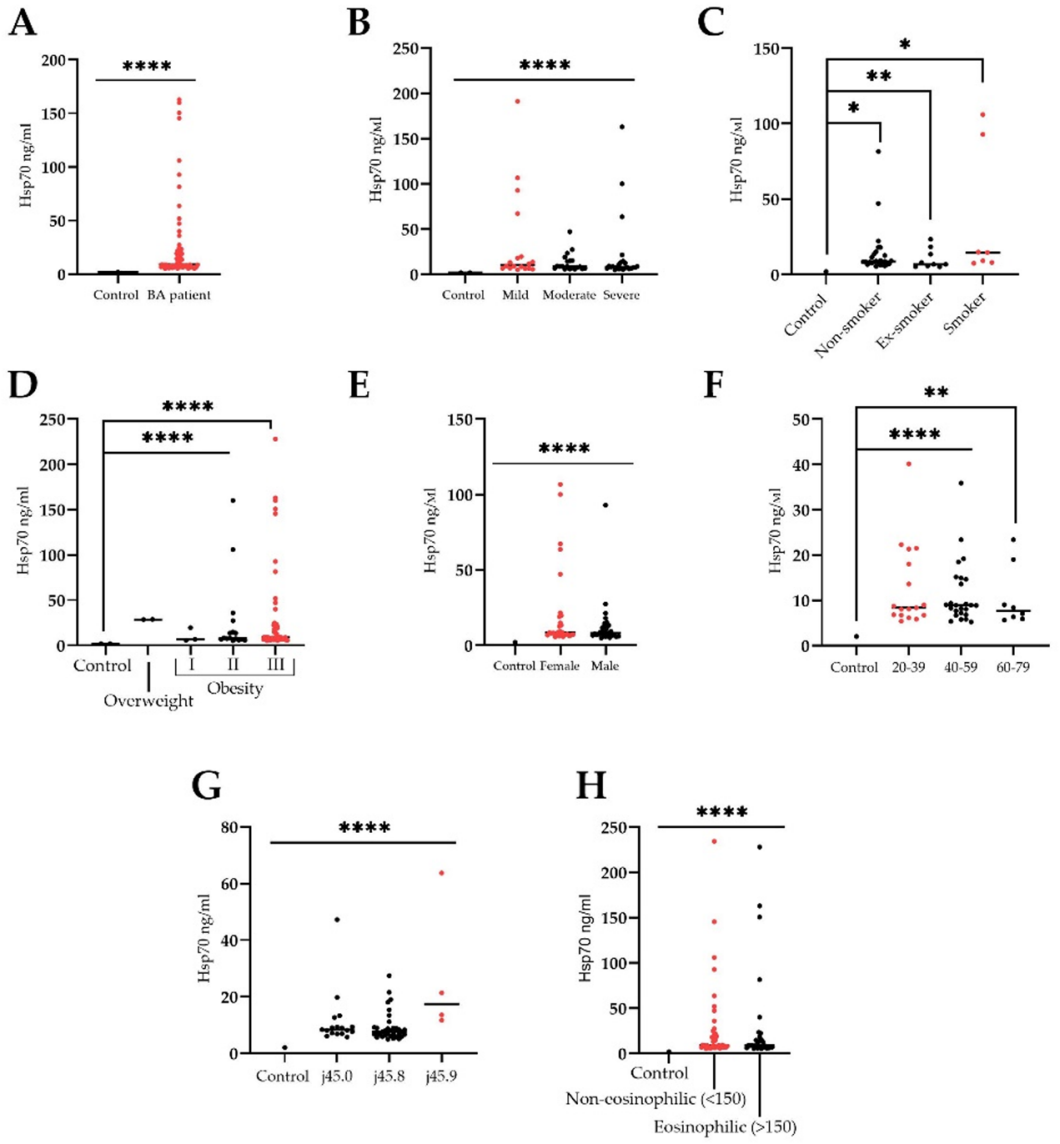
Evaluation of the serum Hsp70 (ng/ml) from healthy volunteers and bronchial asthma patients. (**A**) Comparison of circulating Hsp70 between healthy donors (*n* = 78) and asthmatic patients (*n* = 78). (**B**) Comparison of circulating Hsp70 between healthy volunteers and asthmatic patients of different severity. (**C**) Correlations of Hsp70 levels by smoking (*n* = 42). (**D**) Correlations of Hsp70 levels by BMI (body mass index) (*n* = 73). (**E**) Correlation of serum Hsp70 level by patients’ gender (*n* = 78). (**F**) Correlations of Hsp70 levels in different age groups of patients (*n* = 78). (**G**) Correlations of Hsp70 levels by BA phenotype (*n* = 56). (**H**) Serum Hsp70 content in eosinophilic and non-eosinophilic BA patients. To compare between the groups the Wilcoxon signed rank test was employed. Data is presented as M (mean) ± SE (standard error). Significant differences identified by the Wilcoxon signed rank test are shown as *p < 0.05, ** *p* < 0.01, ***p < 0.001, ****p < 0.0001.

The study of the circulating chaperone HSP70 content in the blood serum of healthy volunteers and patients with bronchial asthma is presented in Fig. 1. In patients with all degrees of asthma severity, the serum HSP70 level was significantly elevated constituting 31.2 ng/ml (5.2–163 ng/ml) as compared to the control group of 2.1 ± 0.21 ng/ml (*p* < 0.001) (Fig. 1A). Further analysis revealed that, compared with the control group, the serum HSP70 level was significantly higher in patients with mild asthma 33.3 ng/ml (5.12–191.2 ng/ml)); *n* = 18; *p* < 0.001), in patients with moderate asthma – 12.4 ng/ml (5.6–41.57 ng/ml)); *n* = 20; *p* < 0.001) and severe asthma – 21.5 ng/ml (4.9–163 ng/ml)); *n* = 24; *p* < 0.001). No significant difference was found when comparing protein content between asthma of different degrees (Fig. 1B). A subsequent analysis comparing the protein level with the sex and age of the patients also did not reveal a statistically significant difference, although women showed a slight increase in the level of serum HSP70 compared to men, constituting 26.3 ng / ml [5.3; 44.1] ng / ml and 23.5 ng / ml [4.94; 23.33] ng / ml), respectively (Fig. 1C, D). Additionally, we detected a strong correlation between the smoking and Hsp70 content in BA patients (Mann-Whitney test, *p* > 0.001) (Fig. 1E), but there was no correlation with BMI (body mass index) (Fig. 1F). Subsequent analysis of the correlation of the Hsp70 serum level and ICS treatment showed that administration of corticosteroids reduced the chaperone levels in BA patients which constituted 15.1 ng/ml (*p* < 0.001). Further analysis of the protein content in different clinical phenotypes of asthma did not reveal any significant differences (Fig. 1G). Thus, in bronchial asthma with a predominant allergic component (J45.0) the serum chaperone constituted 17.54 ng/ml ([5.79; 23.35] ng/ml), in conditionally mixed bronchial asthma (J45.8) – 26.73 ng / ml (4.94; 27.46 ng / ml) and in conditionally unspecified bronchial asthma (J45.9) – 60.31 ng / ml (11.73; 51.98 ng / ml). Evaluation of the eosinophilic asthma patients showed a higher Hsp70 serum content as compared with non-eosinophilic asthma patients, constituting 27.5 ng / ml (5.3–228.3 ng / ml) and 32.5 ng / ml (5.2–234.9 ng / ml), respectively (Fig. 1H).

**Fig. 2:**
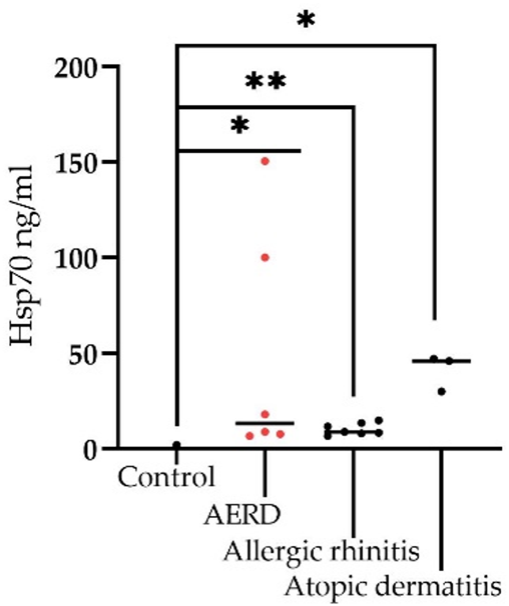
Evaluation of the serum Hsp70 (ng/ml) in bronchial asthma patients with comorbidities. Comparison of circulating Hsp70 in asthmatic patients with concomitant AERD (aspirin-exacerbated respiratory disease), allergic rhinitis, atopic dermatitis. To compare between the groups the Wilcoxon signed rank test was employed. Data is presented as M (mean) ± SE (standard error). Significant differences identified by the Wilcoxon signed rank test are shown as *p < 0.05, ** *p* < 0.01, ***p < 0.001, ****p < 0.0001.

At the next step, we analyzed the serum protein level in asthmatic patients depending on comorbidity (Fig. 2). It turned out that in the presence of concomitant AERD (aspirin-exacerbated respiratory disease), allergic rhinitis and atopic dermatitis, the HSP70 level significantly increased compared to other comorbidities, constituting 473.9 ng / ml (350.8; 597.1 ng / ml), 444.3 ng / ml (307.0; 693.0 ng / ml), and 493.7 ng / ml (374.4; 713.1 ng/ml), respectively (Fig. 2).

### Correlation analysis of the serum Hsp70 levels with Immunoglobulin IgE

**Fig. 3:**
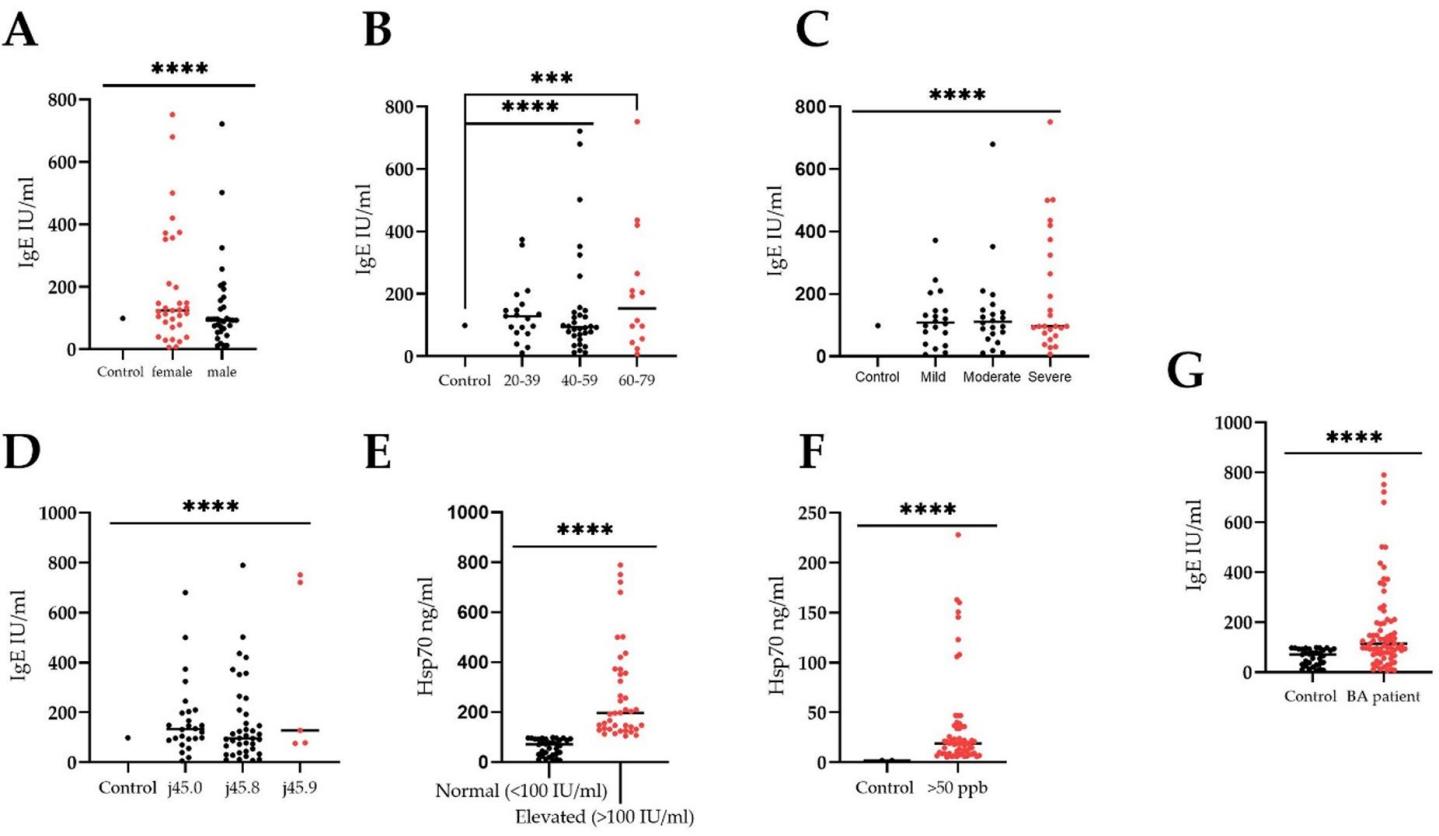
Evaluation of the serum Hsp70 (ng/ml) from bronchial asthma patients and immunoglobulin IgE. Correlations between IgE levels and **(A)** gender and **(B)** age of patients BA with allergic phenotype (J45.0) (*n* = 60). **(C)** Correlation between IgE level and severity of asthma (*n* = 60). **(D)** Correlations between IgE levels and BA (*n* = 60). **(E)** Correlation graph of Hsp70 level and IgE level (*n* = 60). **(F)** Correlations between Hsp70 and FeNO levels. (**G)** Correlations between IgE levels in normal and in bronchial asthma patients. To compare between the groups the Wilcoxon signed rank test was employed. Data is presented as M (mean) ± SE (standard error). Significant differences identified by the Wilcoxon signed rank test are shown as *p < 0.05, ** *p* < 0.01, ***p < 0.001, ****p < 0.0001.

At the next stage, we analyzed the serum immunoglobulin IgE content in asthmatic patients, which can be used as a diagnostic and prognostic marker^38,39^. BA patients had an elevated IgE content compared to the normal level (Fig. 3). At the same time, there was no statistically significant difference in the antibody level depending on the sex, age of patients or type of asthma, severity of asthma, although women had a higher content compared to men, constituting 189.1 (6.8–751.6) and 134.05 (6–191.2) ng / ml, respectively (*p* < 0.05) (Fig. 3A). Further correlation analysis showed that the level of circulating HSP70 correlated with the IgE level (Fig. 3D). Thus, at an IgE level of < 100 IU/ml, the chaperone content was 6–97.2 ng / ml, and with a further increase in the antibody content to > 100IU/ml, an increase in protein was also noted, constituting 105–789.5 ng/ml. Additionally, correlation analysis showed a dependence between the increased level of circulating HSP70 and FeNO level (> 50 ppb) constituting 43.5 ng/ml (5.2–228.1).

### Correlation analysis of the serum Hsp70 levels with cytokines and other markers

**Fig. 4:**
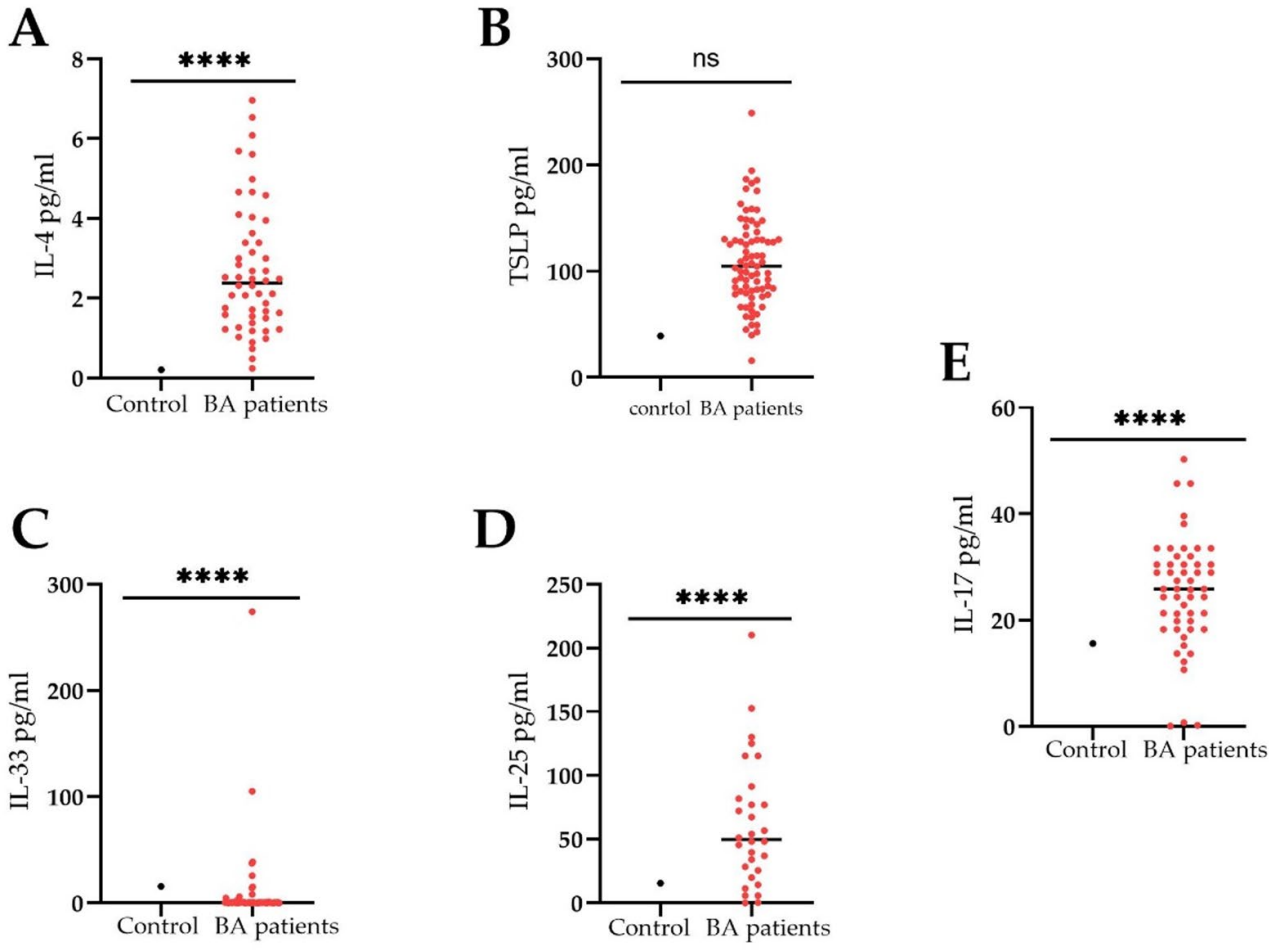
Evaluation of the serum cytokines (pg/ml) from bronchial asthma patients. Comparison of circulating **(A)** IL-4 (*n* = 52), **(B)** TSLP (*n* = 78), **(C)** IL-33 (*n* = 76), **(D)** IL-25 (*n* = 30) and **(E)** IL-17 (*n* = 52) between control and asthmatic patients. To compare between the groups the Wilcoxon signed rank test was employed. Data is presented as M (mean) ± SE (standard error). Significant differences identified by the Wilcoxon signed rank test are shown as *p < 0.05, ** *p* < 0.01, ***p < 0.001, ****p < 0.0001, ns - not significant.

The next step was to study the cytokine content (IL-4, IL-17, IL-25, IL-33, TSLP) in the blood serum of BA patients as well as FeNO. It turned out that the levels were significantly elevated compared to the control reference values. Thus, for IL-4, IL-17, IL-25, IL-33 cytokines the levels constituted 3.4 [0.2; 6.9] pg/ml, 4.8 [0.1; 5.4] pg/ml, 13.6 [0.06; 50.3] pg/ml, 39.9 [0.02; 210] pg/ml, 78 [0.1; 74.2] pg/ml, 10.1 [2.7; 148.4] pg/ml, respectively. The analysis did not reveal a reliable correlation between the level of HSP70 and the following analyzed markers (Fig. 4). However, a strong correlation was found between the level of chaperone and TSLP (Fig. 4B).

### Correlation analysis of the serum Hsp70 levels with lung function

**Fig. 5:**
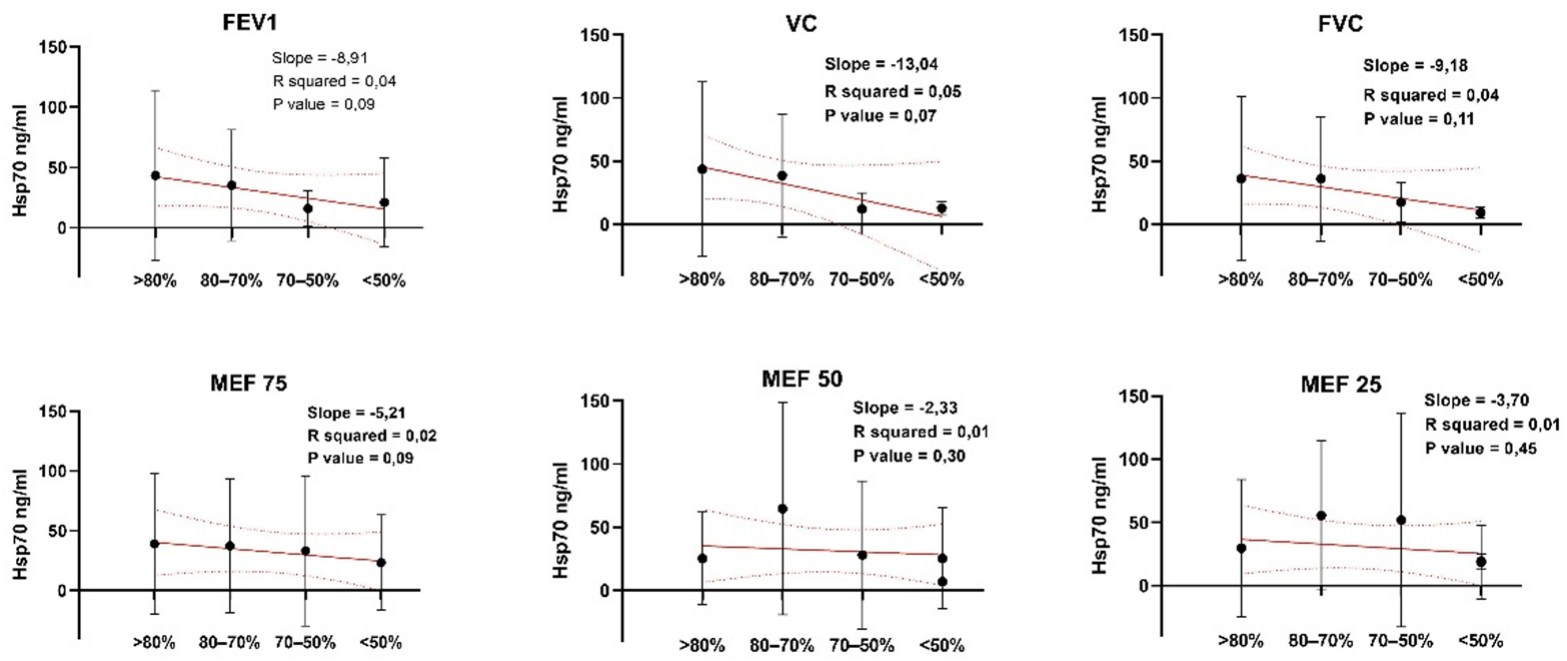
The relationship between HSP70 serum concentration and lung function parameters. Comparison of Hsp70 level during (**A**) FEV1, (**B**) VC, (**C**) FVC, (**D**) MEF 75, (**E**) MEF 50 and (**F**) MEF 25 measurements. Data is presented as M (mean) ± SE (standard error), the linear regression (red lines) with 95% confidence intervals (dashed lines) is built to demonstrate the correlation between HSP70 serum concentration and lung function parameters. The parameters of the linear model (Slope, R squared, P value) are indicated on each plot.

Circulating HSP70 levels in serum in all studied subjects showed significant negative correlation with lung function parameters such as FEV1, VC, FVC, FEF 75 (Fig. 5).

**Fig. 6:**
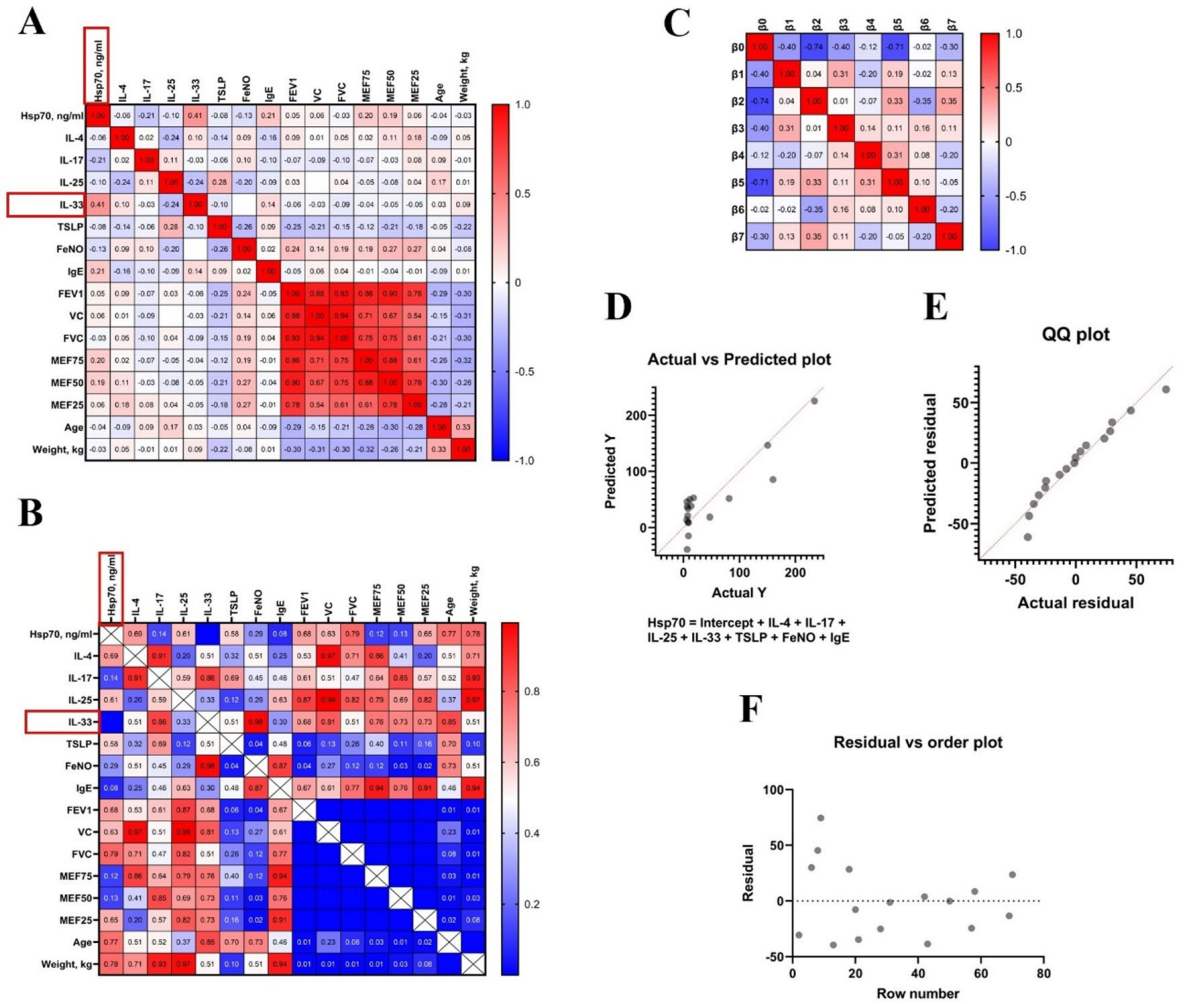
Results of multiple regression model calculation. (**A**) Heat maps showing Pearson correlations for the studied parameters and corresponding P values (**B**). Red bounding boxes indicate a significant interaction (*p* < 0.05). (**C**) Parameter covariance: β0 – Intercept, β1 – IL-4, β2 – IL-17, β3 – IL-25, β4 – IL-33, β5 – TSLP, β6 – FeNO, β7 – IgE. (**D**) Visualization of the performance of a regression model. (**E**) QQ plot for visualizing the normality of the distribution of residues, (**F**) Residual vs. order plot for checking the assumption that the residuals are independent of each other.

In order to evaluate the relationship between one dependent variable (Hsp70 level) and several independent variables (IL-4, IL-17, IL-25, IL-33, TSLP, FeNO, Ige) we used multiple linear regression. The resulting model (R squared 0.784) demonstrates the presence of a strong positive statistically significant correlation between the IL-33 and Hsp70 level (Fig. 6).

## Discussion

The obtained results showed an increased content of circulating serum protein HSP70 in asthmatic patients (Fig. 1). It is worth noting that we did not find a significant difference in the level of chaperone depending on the severity of the course of BA in patients, as well as on gender, age, phenotype of the disease, which is consistent with previously published studies^24,28^. At the same time, we found a strong positive correlation (*p* = 0.02) between the content of HSP70 and smoking of BA patients (Fig. 1C). Previously, several studies have shown that smoking induces the expression of HSP70 as a response to the toxic effects of smoking^40–43^. Thus, in the study of Rumora et al. it was demonstrated that cigarette smoke extract (CSE) and extracellular heat shock protein 70 (eHsp70) (or their combinations) induce ATP release and NLRP3 inflammasome activation in monocytic cells (monocyte-derived macrophages (MDMs) and THP-1) as well as in bronchial epithelial (NCI-H292, 16HBE and NHBE) cells that could lead to inflammation mediated by NLRP3 inflammasomes^43^. Presumably, in asthmatic patients, also with damage to the epithelium of the airways, a compensatory increase in the expression and secretion of the chaperone is observed, however, this hypothesis requires further confirmation. Among the treatment agents applied for management of BA patients we observed a positive correlation between serum Hsp70 content and glucocorticosteroids, when the administration of corticosteroids resulted in significantly reduced levels of chaperone (15.1 ng/ml). This data is in line with previously published data by Bertorelli et al. who showed that fluticasone propionate treatment in patients down-regulated the HLA-DR and Hsp70 expression in BAL and bronchial epithelium^44^. In another study by Wadekar et al. administration of glucocorticoid agonist (dexamethasone) caused inhibition of the heat shock response mediated by heat shock transcription factor 1 (HSF1)^45^.

Hsp70 levels showed a negative correlation (FEV1, Pearson *r* = -0.88, R² = 0.77, *p* = 0.11; VC, Pearson *r* = -0.92, R² = 0.85, *p* = 0.07; FVC, Pearson *r* = -0.95, R² = 0.89, *p* = 0.05) with lung function parameters (Fig. 5). Hsp70 was reliably lower in asthmatics with a “fixed” bronchial structure, which, as we believe, is observed as a result of structural outfits and remodeling of the lower respiratory tract, induced by pro-inflammatory cytokines. Additionally, we detected higher levels of Hsp70 and better FEV-1 values in group of patients with mild asthma (Fig. 5). In a recent work by Schroeder et al. it was proposed that in the chronic disorders characterized by persistent low-grade inflammation the heat shock response (HSR) is gradually suppressed^1^. Partly, this phenomenon could be explained by activation of NOD-like receptor pyrin domain-containing protein-3 inflammasome which in turn influences heat shock transcription factor-1 mRNA expression^46^.

The cytokine storm and HSPs in the wall of the bronchial tree must be adjusted with targeted drugs at the earlier stages of epithelial inflammation, already with asthma of medium-treacherous and, of course, severe course. Biological therapy at the stage of “reversible” obstruction might allow to fully control the inflammatory process in a bronchial tree, preventing the migration of fibroblasts with subsequent irreversible pathophysiological remodeling.

In our work, we determined the presence of circulating HSP70 using a commercial ELISA test kit. It is worth noting that this kit evaluates the content of the dissolved form of protein in biological environments (e.g., serum, plasma, etc.), while it does not take into account the presence of protein, for example, in the composition of extracellular vesicles (exosomes, microvesicles). It is known that immune system cells, as well as tumor cells, are capable of secreting extracellular vesicles along with the soluble form of protein, which also contain the chaperone HSP70 (both directly in the plasma membrane and inside the vesicles themselves)^5,47^. To evaluate the entire pool of extracellular protein, methods were proposed that showed a higher presence of chaperone both in the dissolved form and in the composition of exosomes compared to conventional kits^10,48^. However, the question of the biological function of various forms (dissolved, in vesicles) of the HSP70 protein still remains open – do these functions coincide or is the effect different? That is why in this study we focused on the dissolved form of the protein in serum.

Subsequent correlation analysis with cytokines (i.e., IL-4, IL-17, IL-25) and biochemical markers (FeNO) did not reveal a direct correlation between the HSP70 level and the molecules under study, with the exception of TSLP and IL-33. TSLP protein was previously described as one of the important molecules in the induction of proinflammatory reactions in mucous membranes mediated by the Th2-cell immune response, essentially acting as an alarmin^49–52^. Furthermore, we reported a correlation between IL-33 and Hsp70 (Pearson *r* = 0.41, *p* = 0.002) (Fig. 5). In a recent study by Osorio et al. the authors showed that chaperone directly interacts with IL-33d34, recruiting this cytokine to a vesicular compartment thus enhancing its stability upon secretion. In addition to the correlation with the TSLP and IL-33 levels, we found a strong positive correlation with immunoglobulin IgE (Pearson *r* = 0.99, R² = 0.99, *p* < 0,0001) (Fig. 3), which is also a prognostic and diagnostic marker of allergic reactions^39,53,54^. Furthermore, the level of HSP70 was also statistically significantly increased in eosinophilic asthma, which may also indicate the role of the protein in the Т2 response. Apparently, HSP70 in asthmatic patients acts as an alarmin, which, along with other Т2-mediated molecules, enhances the inflammatory response of the body, thereby participating in the pathogenesis of bronchial asthma^19^. Indeed, as was shown in the recent study by Hlapčić et al. Toll-like receptor 2 (TLR-2) and Hsp70 genes were associated with a pathogenesis of COPD^55^. In this regard, it is not surprising that the level of serum HSP70 was increased in asthma patients complicated by such Т2-associated comorbidities as AERD, allergic rhinitis and atopic dermatitis (Fig. 2).

The current study has several limitations. Firstly, we did not investigate the Hsp70 and interleukins in the induced sputum of patients since the technique of induced sputum analysis is not available in ordinary clinical practice and it may not be obtained in individual patients on the one hand, and on the other hand, it was absent in healthy volunteers. The second restriction of this study lies in the small cohort of the studied patients, and for further study it is recommended to use a larger number of patients, especially since Hsp70 is known to increase in many conditions besides asthma. And the third restriction of the study, in our opinion, is the use of inhalation glucocorticosteroids (IGCS) by patients.

It can be assumed that a decrease in the chaperone level can also lead to a decrease in the inflammatory immune response, which, in turn, can slow down the course of BA. This assumption is supported by a number of studies in which the authors suppressed the function of another representative of the heat shock protein family – HSP90. Thus, in preclinical studies it was shown that inhibition of HSP90 by geldanamycin led to a significant cessation of inflammation and remodeling of the respiratory epithelium in animals^56–60^. Indeed, in the study of Pezzulo et al. inhibition of HSP90 by geldanamycin reverted IL-17-induced goblet cell metaplasia in vivo^60^. Furthermore, as was demonstrated by Yombo et al. in WT and Hsp70 double-knockout (Hsp70.1/0.3^−/−^) mice that were sensitized and intratracheally challenged with *Schistosoma mansoni* soluble egg antigens (SEAs) to induce airway inflammation in the lungs mediated by Th2 responses^61^. The decrease of protein Hsp70 resulted in a significant reduction in airway inflammation accompanied by goblet cell hyperplasia, decrease in eosinophilic infiltration and Th2 cytokine production (IL-4, IL-5, and IL-13)^61^.

## Conclusions

The serum HSP70 content was significantly increased in patients with bronchial asthma, while the protein level positively correlates with such an aggravating factor as smoking in patients. Subsequent analysis revealed a correlation between the content of chaperones and other alarmin molecules – TSLP, immunoglobulin IgE, which may reflect the participation of the protein in the pathogenesis of bronchial asthma mediated by the Т2-immunological response. In conclusion, subsequent studies with a larger sample size of patients will help to determine the role of the HSP70 protein in the pathogenesis of bronchial asthma, as well as the possibility of using the protein as a diagnostic and prognostic marker.

## Materials and methods

### Patients

In accordance with the goals and objectives of this work, a survey was conducted on 78 patients (37 women and 41 men aged 18 to 65 years, average age 45 years) with a verified diagnosis of asthma of any severity and control, receiving basic therapy, the volume of which corresponded to their severity in accordance with the recommendations of the Global Initiative for Asthma (GINA) [Global Initiative for Asthma. Global Strategy for Asthma Management and Prevention, 2024. Update May 2024. Available from: www.ginasthma.org]. Patients included in the study entered the inpatient treatment in the pulmonological department, as well as on an outpatient intake. The control group (CG) included 78 healthy volunteers with a negative allergological history according to the results of tests for allergic diseases (Table 1). All participants were informed about the study, took part in it voluntarily and signed an informed consent to participate in this study.

The study included patients aged 18 years and older with clinical manifestations of bronchial asthma (BA) of varying severity and control according to the GINA 2024 criteria [Global Initiative for Asthma. Global Strategy for Asthma Management and Prevention, 2024. Update May 2024. Available from: www.ginasthma.org]. Verification of the diagnosis of BA was carried out on the basis of clinical and anamnestic data, as well as the results of additional research methods in accordance with current clinical guidelines. Patients with a severe form of bronchial asthma at the time of blood fence did not receive biological drugs (e.g., omalizumab, mepolizumab, etc.).

All patients underwent the following procedures: filling out questionnaires on the history of smoking, drug allergies, other allergic diseases, physical examination, lung spirometry, blood sampling to assess peripheral blood cells and HSP70 and cytokine levels.

According to the number of eosinophils in the peripheral blood, patients with asthma were divided into a group with eosinophilic (> 150 cells/µl) asthma (*n* = 43) and non-eosinophilic (< 150 cells/µl) asthma (*n* = 28)^62^.

Patients assessed by body mass index (BMI) were divided into groups with obesity (BMI ≥ 30 kg/m^2^) and asthma (*n* = 20) and without obesity (BMI < 30 kg/m^2^) and asthma (*n* = 58). BMI was calculated using the formula: BMI = weight (kg)/height (m^2^) for all individuals^63^.

We also stratified patients by asthma severity as defined by the GINA 2024 criteria, and thus divided patients into mild, moderate, and severe, depending on the need for inhaled corticosteroids (ICS). Patients with mild asthma were treated with a short-acting bronchodilator - short-acting β2 agonist (SABA) as needed or low-dose ICS (≤ 250 µg fluticasone equivalent); moderate asthmatics – with low-dose ICS with a long-acting β2 agonist (LABA) (Step 3 treatment). Severe asthmatics were patients who required a therapy volume corresponding to GINA step 4 (high-dose ICS (> 500 µg fluticasone equivalent) and a LABA or tiotropium.

The severity of asthma in patients receiving treatment is assessed retrospectively and is determined by the amount of therapy (conducted over the previous few months) required to achieve symptom control^64–66^.

### Inclusion criteria

Inclusion criteria: men or women aged 18 years and older with a verified diagnosis of asthma of any severity and control; written informed consent. Of the comorbide diseases, patients with T2-associated diseases (AR (allergic rhinitis), AD (atopic dermatitis), CPRS (chronic polypoic rhinosinusitis) were also included into the study.

### Exclusion criteria

Exclusion criteria: age under 18 years; no informed consent; failure to meet inclusion/non-inclusion criteria. Patients with other inflammatory diseases, the presence of concomitant chronic bronchial pathology; The patient’s extremely serious condition, requiring resuscitation meal; The use of systemic therapy with oral corticosteroids, cyclosporine, biological drugs.

### Lung function assessment

Clinical manifestations of bronchial asthma are attacks of broncho-obstruction leading to airway obstruction. To assess the severity of broncho-obstruction, all patients underwent external respiratory function (ERF). Spirometry was performed in compliance with the recommendations for conducting and interpreting the results of lung function testing, using the Spirosft-3000 device (Funuda Densh Co., Ltd. Japan) and included the following parameters: vital capacity of the lungs (VC), forced expiratory volume in 1 s (FEV1), forced expiratory flows at the level of large, medium, small bronchi (FEV25, FEV50, FEV75) as a percentage of the expected values. All subjects were asked to avoid using SABA for at least 8 h before the test. Patients underwent spirometry in a sitting position, using a nose clip. The degree of obstruction was assessed by functional indices (FEV1) before the bronchodilator. Functional indices: intermittent FEV1 ≥ 80% predicted; mild FEV1 ≥ 80% predicted; moderate FEV1 60–80% predicted; severe FEV1 ≤ 60% predicted.

### Blood sample Preparation

In order to clarify the diagnosis and select the therapeutic tactics, peripheral blood of patients with BA was examined, in which the HSP70 serum level and other markers were determined. For this, 7–8 ml of the patient’s blood was collected in vacutainers (vacuum test tubes for collecting venous blood “UNIVAC” with an anticoagulant) containing tripotassium salt of ethylenediaminetetraacetic acid (K3 EDTA). Blood was collected on an empty stomach from the cubital vein with a disposable needle into a vacuum test tube (the tube cap is red). Venous blood was settled in the test tube at room temperature for 30 min until the clot was completely formed. After clot retraction, the samples were centrifuged at 3000 rpm for 10 min at room temperature. Then, after aliquoting, the separated plasma was collected in 1 ml portions into Eppendorf microcentrifuge tubes, labeled with the department, patient’s full name, collection date, and kept at a temperature of – 80 °C until completely frozen, and then defrosted at room temperature for analysis.

### Detection of cytokines and markers

Cytokines IL-4, IL-17, IL-25, IL-33, and TSLP (ELISA Cloud-Clone Corp, Katy, TX, USA) were detected by the standard sandwich ELISA assay according to the manufacturers protocol. For the quantitative determination of total IgE, the Reagent Kit for the quantitative enzyme immunoassay determination of total IgE in human blood serum “ELISA-total IgE” B-12.2015-02 was used (Alkor Bio Company LLC, St. Petersburg, Russia). FeNOmetry – determination of the fraction of nitric oxide in exhaled air. Nitric oxide (FeNO) is a gas produced by cells involved in the inflammatory process associated with asthma and represents a non-invasive biomarker of inflammation of the respiratory tract which is used to detect eosinophilic inflammation of the respiratory tract and predict reaction on steroids. This is a breathing test used to determine whether there is an inflammatory process in the lungs, which is assessed by the FeNOmetry mechanism. FeNOmetry – using the Bedfont NOBreath nitric oxide analyzer (Bedfont NOBreath (NO) UK), with a set of individual mouthpieces. Sensitivity on the test constituted 5 ppb (parts per billion). FeNO values in adult patients were intended as follows: <25 ppb - low values; >25 ppb – high values (taking into account the clinical situation).

### ELISA assay for Hsp70 serum detection

The Human/Mouse/Rat Total HSP70/HSPA1A DuoSet IC ELISA kit (cat. no. DYC1663-2, R&D Systems, USA) was used to set up the sandwich ELISA.

A 96-well microtiter plate was pre-coated with a capture antibody (cat. no. 841680, R&D Systems, USA) at a concentration of 2.0 µg/ml, 100 µl per well, and left overnight at room temperature. The antibodies were washed three times with wash buffer (cat. no. WA126, R&D Systems, USA). Then 300 µl of blocking buffer (cat. no. DY994, R&D Systems, USA) were added to each well and incubated for 2 h, followed by washing.

The test sample (patient blood plasma) in a volume of 100 µl was diluted in IC Diluent #4 (cat. no. DYC001, R&D Systems, USA) and incubated for 2 h at room temperature, followed by washing. The detection antibody (cat. no. 841681, R&D Systems, USA) was diluted in IC Diluent #1 (cat. no. DY995, R&D Systems, USA) at a concentration of 100 ng/ml, 100 µl were added per well and incubated for 2 h at room temperature. After washing, streptavidin-horseradish peroxidase conjugate (cat. no. cat. no. 890803, R&D Systems, USA) was added to each well for 20 min, followed by washing. Next, the substrate solution (cat. no. DY999, R&D Systems, USA) was added, incubated for 20 min, after which the stop solution (cat. no. DY994, R&D Systems, USA) was added and the absorbance was read at a wavelength of 405 nm using a Varioskan LUX multimode microplate reader (Thermo Fisher Scientific, USA).

### Statistics

To assess the relationship between the Hsp70 level and patient parameters (age, gender, severity and phenotype of the disease, IgE level), the results were collected in a table and statistically processed using GraphPad Prism 9 software (GraphPad Software Inc., USA). The Shapiro-Wilk criteria were used to check the data for normality. When the data were not normally distributed, the nonparametric Wilcoxon signed rank test was used to compare the groups. The data is presented as median values using 95% confidence intervals or as M (mean) ± SE (standard error). In order to show the relationship between the HSP70 concentration in serum and the parameters of the lung function, linear regression was built for four measurement groups corresponding to various levels of lung functions (< 50%, 50–70%, 70–80%, > 80%). Data analysis was performed using the multiple regression method. Heat maps were used to visualize the correlation of Pearson correlations for the studied parameters and corresponding P values. The numerical values of the parameter correlations are presented in Table S1.

## Supplementary Information

Below is the link to the electronic supplementary material.

Supplementary Material 1

## Data Availability

The datasets used and/or analyzed during the current study are available from the corresponding author Maxim Shevtsov and Ella Churyukina on reasonable request.

## Abbreviations

ATP: Adenosine triphosphate
BA: Bronchial asthma
BLAF: Bronchoalveolar lavage fluid
BMI: Body mass index
COPD: Chronic obstructive pulmonary disease
CSE: Cigarette smoke extract
DAMP: Damage-associated molecular pattern molecules
EDTA: Ethylenediaminetetraacetic acid
FEV1: Forced exhalation volume in 1 s
FVC: Accelerated vital capacity of the lungs
HELLP syndrome: Hemolysis, elevated liver enzymes, low platelet count
HMGBM1: High-mobility group box-1
IgE: Immunoglobulin E
IL-25: Interleukin 25
IL-33: Interleukin 33
LL-37: Antimicrobial peptide cathelicidin
LOX-1: Lectin-like oxidized low-density lipoprotein receptor
LRP: Leucine responsive protein
MARCKS: Myristoylated alanine-rich C kinase substrate
NHBE: Normal human bronchial epithelial cells
NLRP3: The inflammasom, Nod-like receptor of the NALP family
S100A8: Calcium-binding protein A8
SEAs: Soluble egg antigens
Th2: Т-helper 2
TLR2: Toll-like receptors 2
TLR4: Toll-like receptors 4
TNFα: Tumor necrosis factorα
TSLP: Thymus-stromal lymphopoietin

## References

[1] Naik, S. R. & Wala, S. M. Inflammation, allergy and asthma, complex immune origin diseases: mechanisms and therapeutic agents. *Recent. Pat. Inflamm. Allergy Drug Discov*. **7**, 62–95 (2013). 23140569

[2] Shevtsov, M. & Multhoff, G. Heat shock Protein-Peptide and HSP-Based immunotherapies for the treatment of cancer. *Front. Immunol.* **7**, 171. 10.3389/fimmu.2016.00171 (2016). 27199993 10.3389/fimmu.2016.00171 PMC4850156

[3] Mayer, M. P. & Bukau, B. Hsp70 chaperones: cellular functions and molecular mechanism. *Cell. Mol. Life Sci.* **62**, 670–684. 10.1007/s00018-004-4464-6 (2005). 15770419 10.1007/s00018-004-4464-6 PMC2773841

[4] Sekhar, A., Rosenzweig, R., Bouvignies, G. & Kay, L. E. Hsp70 biases the folding pathways of client proteins. *Proc. Natl. Acad. Sci. U S A*. **113**, E2794–2801. 10.1073/pnas.1601846113 (2016). 27140645 10.1073/pnas.1601846113 PMC4878499

[5] Guzhova, I. V., Shevtsov, M. A., Abkin, S. V., Pankratova, K. M. & Margulis, B. A. Intracellular and extracellular Hsp70 chaperone as a target for cancer therapy. *Int. J. Hyperth.* **29**, 399–408. 10.3109/02656736.2013.807439 (2013). 10.3109/02656736.2013.807439 23845032

[6] Asea, A. Stress proteins and initiation of immune response: chaperokine activity of hsp72. *Exerc. Immunol. Rev.* **11**, 34–45 (2005). 16385842 PMC1762141

[7] Khandia, R., Munjal, A. K., Iqbal, H. M. N. & Dhama, K. Heat shock proteins: therapeutic perspectives in inflammatory disorders. *Recent. Pat. Inflamm. Allergy Drug Discov*. **10**, 94–104. 10.2174/1872213x10666161213163301 (2017). 27978789 10.2174/1872213X10666161213163301

[8] Thériault, J. R., Mambula, S. S., Sawamura, T., Stevenson, M. A. & Calderwood, S. K. Extracellular HSP70 binding to surface receptors present on antigen presenting cells and endothelial/epithelial cells. *FEBS Lett.* **579**, 1951–1960. 10.1016/j.febslet.2005.02.046 (2005). 15792802 10.1016/j.febslet.2005.02.046

[9] Asea, A. et al. HSP70 stimulates cytokine production through a CD14-dependant pathway, demonstrating its dual role as a chaperone and cytokine. *Nat. Med.* **6**, 435–442. 10.1038/74697 (2000). 10742151 10.1038/74697

[10] Salvermoser, L. et al. Elevated Circulating Hsp70 levels are correlative for malignancies in different mammalian species. *Cell. Stress Chaperones*. **28**, 105–118. 10.1007/s12192-022-01311-y (2023). 36399258 10.1007/s12192-022-01311-y PMC9877270

[11] Rodríguez-Iturbe, B., Johnson, R. J. & Sánchez-Lozada, L. G. Relationship between hyperuricemia, HSP70 and NLRP3 inflammasome in arterial hypertension. *Arch. Cardiol. Mex*. **93**, 458–463. 10.24875/acm.22000174 (2023). 37972372 10.24875/ACM.22000174

[12] Kasperkiewicz, M. & Tukaj, S. Targeting heat shock proteins 90 and 70: A promising remedy for both autoimmune bullous diseases and COVID-19. *Front. Immunol.* **13**, 1080786. 10.3389/fimmu.2022.1080786 (2022). 36591225 10.3389/fimmu.2022.1080786 PMC9797581

[13] Tukaj, S. & Sitko, K. Heat shock protein 90 (Hsp90) and Hsp70 as potential therapeutic targets in autoimmune skin diseases. *Biomolecules* **12**10.3390/biom12081153 (2022). 10.3390/biom12081153 PMC9405624 36009046

[14] Fouani, M. et al. Heat shock proteins alterations in rheumatoid arthritis. *Int. J. Mol. Sci.* 23. 10.3390/ijms23052806 (2022). 10.3390/ijms23052806 PMC8911505 35269948

[15] Thorsteinsdottir, J. et al. Overexpression of cytosolic, plasma membrane bound and extracellular heat shock protein 70 (Hsp70) in primary glioblastomas. *J. Neurooncol*. **135**, 443–452. 10.1007/s11060-017-2600-z (2017). 28849427 10.1007/s11060-017-2600-z

[16] Villafan-Bernal, J. R. et al. Relevant serum Endoplasmic reticulum stress biomarkers in type 2 diabetes and its complications: A systematic review and Meta-Analysis. *Antioxid. (Basel)*. 13. 10.3390/antiox13121564 (2024). 10.3390/antiox13121564 PMC11673038 39765892

[17] Faisal, S. et al. Diagnostic and prognostic risk assessment of heat shock protein HSPA1B rs2763979 gene variant in asthma. *Genes (Basel)*. 13. 10.3390/genes13122391 (2022). 10.3390/genes13122391 PMC9778050 36553658

[18] Yang, M. et al. Plasma antibodies against heat shock protein 70 correlate with the incidence and severity of asthma in a Chinese population. *Respir Res.* **6**, 18. 10.1186/1465-9921-6-18 (2005). 15710045 10.1186/1465-9921-6-18 PMC549531

[19] Shevchenko, M., Servuli, E., Albakova, Z., Kanevskiy, L. & Sapozhnikov, A. The role of heat shock protein 70 kDa in asthma. *J. Asthma Allergy*. **13**, 757–772. 10.2147/jaa.S288886 (2020). 33447061 10.2147/JAA.S288886 PMC7801907

[20] Fang, S. et al. MARCKS and HSP70 interactions regulate mucin secretion by human airway epithelial cells in vitro. *Am. J. Physiol. Lung Cell. Mol. Physiol.* **304**, L511–518. 10.1152/ajplung.00337.2012 (2013). 23377348 10.1152/ajplung.00337.2012 PMC3625989

[21] Prado, N. et al. Exosomes from Bronchoalveolar fluid of tolerized mice prevent allergic reaction. *J. Immunol.* **181**, 1519–1525. 10.4049/jimmunol.181.2.1519 (2008). 18606707 10.4049/jimmunol.181.2.1519

[22] Hinchado, M. D., Giraldo, E. & Ortega, E. Adrenoreceptors are involved in the stimulation of neutrophils by exercise-induced Circulating concentrations of Hsp72: cAMP as a potential intracellular danger signal. *J. Cell. Physiol.* **227**, 604–608. 10.1002/jcp.22759 (2012). 21448922 10.1002/jcp.22759

[23] Wheeler, D. S. et al. Extracellular Hsp72, an endogenous DAMP, is released by virally infected airway epithelial cells and activates neutrophils via Toll-like receptor (TLR)-4. *Respir Res.* **10**, 31. 10.1186/1465-9921-10-31 (2009). 19405961 10.1186/1465-9921-10-31 PMC2679007

[24] Tamási, L. et al. Increased Circulating heat shock protein 70 levels in pregnant asthmatics. *Cell. Stress Chaperones*. **15**, 295–300. 10.1007/s12192-009-0143-8 (2010). 19777374 10.1007/s12192-009-0143-8 PMC2866990

[25] Molvarec, A. et al. Circulating heat shock protein 70 (HSPA1A) in normal and pathological pregnancies. *Cell. Stress Chaperones*. **15**, 237–247. 10.1007/s12192-009-0146-5 (2010). 19821156 10.1007/s12192-009-0146-5 PMC2866993

[26] Molvarec, A. et al. Association of increased serum heat shock protein 70 and C-reactive protein concentrations and decreased serum alpha(2)-HS glycoprotein concentration with the syndrome of hemolysis, elevated liver enzymes, and low platelet count. *J. Reprod. Immunol.* **73**, 172–179. 10.1016/j.jri.2006.07.002 (2007). 17023052 10.1016/j.jri.2006.07.002

[27] Molvarec, A. et al. Serum heat shock protein 70 levels in relation to Circulating cytokines, chemokines, adhesion molecules and angiogenic factors in women with preeclampsia. *Clin. Chim. Acta*. **412**, 1957–1962. 10.1016/j.cca.2011.06.042 (2011). 21756887 10.1016/j.cca.2011.06.042

[28] Hou, C. et al. Increased heat shock protein 70 levels in induced sputum and plasma correlate with severity of asthma patients. *Cell. Stress Chaperones*. **16**, 663–671. 10.1007/s12192-011-0271-9 (2011). 21643870 10.1007/s12192-011-0271-9 PMC3220390

[29] Huang, X. et al. Differential DAMP release was observed in the sputum of COPD, asthma and asthma-COPD overlap (ACO) patients. *Sci. Rep.* **9**, 19241. 10.1038/s41598-019-55502-2 (2019). 31848359 10.1038/s41598-019-55502-2 PMC6917785

[30] Rincon, M. & Irvin, C. G. Role of IL-6 in asthma and other inflammatory pulmonary diseases. *Int. J. Biol. Sci.* **8**, 1281–1290. 10.7150/ijbs.4874 (2012). 23136556 10.7150/ijbs.4874 PMC3491451

[31] Gurczynski, S. J. & Moore, B. B. IL-17 in the lung: the good, the bad, and the ugly. *Am. J. Physiol. Lung Cell. Mol. Physiol.* **314**, L6–l16. 10.1152/ajplung.00344.2017 (2018). 28860146 10.1152/ajplung.00344.2017 PMC6048455

[32] Santus, P., Saad, M., Damiani, G., Patella, V. & Radovanovic, D. Current and future targeted therapies for severe asthma: managing treatment with biologics based on phenotypes and biomarkers. *Pharmacol. Res.* **146**, 104296. 10.1016/j.phrs.2019.104296 (2019). 31173886 10.1016/j.phrs.2019.104296

[33] Borish, L. The immunology of asthma: asthma phenotypes and their implications for personalized treatment. *Ann. Allergy Asthma Immunol.* **117**, 108–114. 10.1016/j.anai.2016.04.022 (2016). 27499537 10.1016/j.anai.2016.04.022 PMC4977430

[34] Celebi Sozener, Z. et al. Epithelial barrier hypothesis: effect of the external exposome on the Microbiome and epithelial barriers in allergic disease. *Allergy* **77**, 1418–1449. 10.1111/all.15240 (2022). 35108405 10.1111/all.15240 PMC9306534

[35] Baxi, S. N. & Phipatanakul, W. The role of allergen exposure and avoidance in asthma. *Adolesc. Med. State Art Rev.* **21**, 57–71 (2010). viii-ix. 20568555 PMC2975603

[36] Brusselle, G. & Bracke, K. Targeting immune pathways for therapy in asthma and chronic obstructive pulmonary disease. *Ann. Am. Thorac. Soc.* **11** (Suppl 5), 322–328. 10.1513/AnnalsATS.201403-118AW (2014). 10.1513/AnnalsATS.201403-118AW 25525740

[37] Brusselle, G. G., Maes, T. & Bracke, K. R. Eosinophils in the spotlight: eosinophilic airway inflammation in nonallergic asthma. *Nat. Med.* **19**, 977–979. 10.1038/nm.3300 (2013). 23921745 10.1038/nm.3300

[38] Obaidi, A. A., Mohamed Al Samarai, A. H., Yahya Al Samarai, A. G., Al, A. K. & Janabi, J. M. The predictive value of IgE as biomarker in asthma. *J. Asthma*. **45**, 654–663. 10.1080/02770900802126958 (2008). 18951256 10.1080/02770900802126958

[39] Gevaert, P., Wong, K., Millette, L. A. & Carr, T. F. The role of IgE in upper and lower airway disease: more than just Allergy! *Clin. Rev. Allergy Immunol.* **62**, 200–215. 10.1007/s12016-021-08901-1 (2022). 34536215 10.1007/s12016-021-08901-1 PMC8818003

[40] Anbarasi, K., Kathirvel, G., Vani, G., Jayaraman, G. & Shyamala Devi, C. S. Cigarette smoking induces heat shock protein 70 kDa expression and apoptosis in rat brain: modulation by bacoside A. *Neuroscience* **138**, 1127–1135. 10.1016/j.neuroscience.2005.11.029 (2006). 16472926 10.1016/j.neuroscience.2005.11.029

[41] Ospelt, C. et al. Smoking induces transcription of the heat shock protein system in the joints. *Ann. Rheum. Dis.* **73**, 1423–1426. 10.1136/annrheumdis-2013-204486 (2014). 24550170 10.1136/annrheumdis-2013-204486

[42] Satta, S. et al. A Nrf2-OSGIN1&2-HSP70 axis mediates cigarette smoke-induced endothelial detachment: implications for plaque erosion. *Cardiovasc. Res.* **119**, 1869–1882. 10.1093/cvr/cvad022 (2023). 36804807 10.1093/cvr/cvad022 PMC10405570

[43] Rumora, L., Somborac-Bačura, A., Hlapčić, I., Hulina-Tomašković, A. & Rajković, M. G. Cigarette smoke and extracellular Hsp70 induce secretion of ATP and differential activation of NLRP3 inflammasome in monocytic and bronchial epithelial cells. *Cytokine* **135**, 155220. 10.1016/j.cyto.2020.155220 (2020). 32736335 10.1016/j.cyto.2020.155220

[44] Bertorelli, G. et al. Heat shock protein 70 upregulation is related to HLA-DR expression in bronchial asthma. Effects of inhaled glucocorticoids. *Clin. Exp. Allergy*. **28**, 551–560. 10.1046/j.1365-2222.1998.00251.x (1998). 9645591 10.1046/j.1365-2222.1998.00251.x

[45] Wadekar, S. A., Li, D. & Sánchez, E. R. Agonist-activated glucocorticoid receptor inhibits binding of heat shock factor 1 to the heat shock protein 70 promoter in vivo. *Mol. Endocrinol.* **18** (3), 500–508. 10.1210/me.2003-0215 (2004). 14673132 10.1210/me.2003-0215

[46] Schroeder, H. T., De Lemos Muller, C. H., Heck, T. G. & Krause, M. Homem de Bittencourt P.I. Heat shock response during the resolution of inflammation and its progressive suppression in chronic-degenerative inflammatory diseases. *Cell. Stress Chaperones*. **29** (1), 116–142. 10.1016/j.cstres.2024.01.002 (2024). 38244765 10.1016/j.cstres.2024.01.002 PMC10939074

[47] Gastpar, R. et al. The cell surface-localized heat shock protein 70 epitope TKD induces migration and cytolytic activity selectively in human NK cells. *J. Immunol.* **172**, 972–980. 10.4049/jimmunol.172.2.972 (2004). 14707070 10.4049/jimmunol.172.2.972

[48] Werner, C. et al. Hsp70 in liquid Biopsies-A Tumor-Specific biomarker for detection and response monitoring in cancer. *Cancers (Basel)*. 10.3390/cancers13153706 (2021). 10.3390/cancers13153706 PMC8345117 34359606

[49] Comeau, M. R. & Ziegler, S. F. The influence of TSLP on the allergic response. *Mucosal Immunol.* **3**, 138–147. 10.1038/mi.2009.134 (2010). 20016474 10.1038/mi.2009.134

[50] Wang, W. L. et al. Thymic stromal lymphopoietin: a promising therapeutic target for allergic diseases. *Int. Arch. Allergy Immunol.* **160**, 18–26. 10.1159/000341665 (2013). 22948028 10.1159/000341665

[51] Ito, T., Liu, Y. J. & Arima, K. Cellular and molecular mechanisms of TSLP function in human allergic disorders–TSLP programs the Th2 code in dendritic cells. *Allergol. Int.* **61**, 35–43. 10.2332/allergolint.11-RAI-0376 (2012). 22189594 10.2332/allergolint.11-RAI-0376 PMC3660852

[52] Cheng, D. T. et al. Thymic stromal lymphopoietin receptor Blockade reduces allergic inflammation in a cynomolgus monkey model of asthma. *J. Allergy Clin. Immunol.* **132**, 455–462. 10.1016/j.jaci.2013.05.011 (2013). 23810153 10.1016/j.jaci.2013.05.011

[53] Eguiluz-Gracia, I., Layhadi, J. A., Rondon, C. & Shamji, M. H. Mucosal IgE immune responses in respiratory diseases. *Curr. Opin. Pharmacol.* **46**, 100–107. 10.1016/j.coph.2019.05.009 (2019). 31220711 10.1016/j.coph.2019.05.009

[54] Rondón, C. et al. IgE test in secretions of patients with respiratory allergy. *Curr. Allergy Asthma Rep.* **18**, 67. 10.1007/s11882-018-0821-7 (2018). 30317418 10.1007/s11882-018-0821-7

[55] Hlapčić, I. et al. Increased HSP70 and TLR2 gene expression and association of HSP70 rs6457452 single nucleotide polymorphism with the risk of chronic obstructive pulmonary disease in the Croatian population. *Diagnostics (Basel)*. 10.3390/diagnostics11081412 (2021). 10.3390/diagnostics11081412 PMC8394658 34441346

[56] Colunga Biancatelli, R. M. L. et al. The heat shock protein 90 Inhibitor, AT13387, protects the Alveolo-Capillary barrier and prevents HCl-Induced chronic lung injury and pulmonary fibrosis. *Cells* **11**10.3390/cells11061046 (2022). 10.3390/cells11061046 PMC8946990 35326496

[57] Colunga Biancatelli, R. M. L., Solopov, P., Gregory, B. & Catravas, J. D. The HSP90 Inhibitor, AUY-922, protects and repairs human lung microvascular endothelial cells from hydrochloric Acid-Induced endothelial barrier dysfunction. *Cells* **10**10.3390/cells10061489 (2021). 10.3390/cells10061489 PMC8232030 34199261

[58] Ye, C. et al. The role of secreted Hsp90α in HDM-induced asthmatic airway epithelial barrier dysfunction. *BMC Pulm Med.* **19**, 218. 10.1186/s12890-019-0938-z (2019). 31747880 10.1186/s12890-019-0938-z PMC6868813

[59] Dong, H. M. et al. Extracellular heat shock protein 90α mediates HDM-induced bronchial epithelial barrier dysfunction by activating RhoA/MLC signaling. *Respir Res.* **18**, 111. 10.1186/s12931-017-0593-y (2017). 28558721 10.1186/s12931-017-0593-y PMC5450201

[60] Pezzulo, A. A. et al. HSP90 inhibitor geldanamycin reverts IL-13- and IL-17-induced airway goblet cell metaplasia. *J. Clin. Invest.* **129**, 744–758. 10.1172/jci123524 (2019). 30640172 10.1172/JCI123524 PMC6355221

[61] Yombo, D. J. K., Mentink-Kane, M. M., Wilson, M. S., Wynn, T. A. & Madala, S. K. Heat shock protein 70 is a positive regulator of airway inflammation and goblet cell hyperplasia in a mouse model of allergic airway inflammation. *J. Biol. Chem.* **294**, 15082–15094. 10.1074/jbc.RA119.009145 (2019). 31431507 10.1074/jbc.RA119.009145 PMC6791332

[62] Oppenheimer, J. et al. Allergic and eosinophilic asthma in the era of biomarkers and biologics: similarities, differences and misconceptions. *Ann. Allergy Asthma Immunol.* **129**, 169–180. 10.1016/j.anai.2022.02.021 (2022). 35272048 10.1016/j.anai.2022.02.021

[63] Peters, U., Dixon, A. E. & Forno, E. Obesity and asthma. *J. Allergy Clin. Immunol.* **141**, 1169–1179. 10.1016/j.jaci.2018.02.004 (2018). 29627041 10.1016/j.jaci.2018.02.004 PMC5973542

[64] Reddel, H. K. et al. An official American thoracic society/European respiratory society statement: asthma control and exacerbations: standardizing endpoints for clinical asthma trials and clinical practice. *Am. J. Respir Crit. Care Med.* **180**, 59–99. 10.1164/rccm.200801-060ST (2009). 19535666 10.1164/rccm.200801-060ST

[65] Taylor, D. R. et al. A new perspective on concepts of asthma severity and control. *Eur. Respir J.* **32**, 545–554. 10.1183/09031936.00155307 (2008). 18757695 10.1183/09031936.00155307

[66] Chung, K. F. et al. International ERS/ATS guidelines on definition, evaluation and treatment of severe asthma. *Eur. Respir J.* **43**, 343–373. 10.1183/09031936.00202013 (2014). 24337046 10.1183/09031936.00202013

