# Supplementary material for: Increased circulating heat shock protein Hsp70 serum levels as a potential biomarker in bronchial asthma patients
Source: Sci Rep. 2025 Nov 18;15:40335. doi: 10.1038/s41598-025-28297-8 (PMC12627502; doi:10.1038/s41598-025-28297-8)
Supplement: Supplementary file 1 — Supplementary Material 1 [file 41598_2025_28297_MOESM1_ESM.docx]

| Parameter estimates | Variable | Estimate | Standard error | 95% confidence interval |  |  |  |  |  |
| --- | --- | --- | --- | --- | --- | --- | --- | --- | --- |
| β0 | Intercept | 57,33 | 70,19 | -101,4 to 216,1 |  |  |  |  |  |
| β1 | B : IL-4 | -5,809 | 5,695 | -18,69 to 7,074 |  |  |  |  |  |
| β2 | C : IL-17 | -1,749 | 1,706 | -5,608 to 2,110 |  |  |  |  |  |
| β3 | D : IL-25 | -0,1568 | 0,2839 | -0,7990 to 0,4854 |  |  |  |  |  |
| β4 | E : IL-33 | 0,8315 | 0,1876 | 0,4071 to 1,256 |  |  |  |  |  |
| β5 | F : TSLP | 0,3371 | 0,2708 | -0,2754 to 0,9496 |  |  |  |  |  |
| β6 | G : FeNO | -0,1341 | 0,448 | -1,148 to 0,8794 |  |  |  |  |  |
| β7 | H : IgE | 0,02961 | 0,01816 | -0,01148 to 0,07070 |  |  |  |  |  |
| Sig. diff. than zero | Variable | \|t\| | P value | P value summary |  |  |  |  |  |
| β0 | Intercept | 0,8169 | 0,4351 | ns |  |  |  |  |  |
| β1 | B : IL-4 | 1,02 | 0,3344 | ns |  |  |  |  |  |
| β2 | C : IL-17 | 1,025 | 0,332 | ns |  |  |  |  |  |
| β3 | D : IL-25 | 0,5522 | 0,5943 | ns |  |  |  |  |  |
| β4 | E : IL-33 | 4,432 | 0,0016 | ** |  |  |  |  |  |
| β5 | F : TSLP | 1,245 | 0,2445 | ns |  |  |  |  |  |
| β6 | G : FeNO | 0,2993 | 0,7715 | ns |  |  |  |  |  |
| β7 | H : IgE | 1,63 | 0,1375 | ns |  |  |  |  |  |
| Goodness of Fit |  |  |  |  |  |  |  |  |  |
| Degrees of Freedom | 9 |  |  |  |  |  |  |  |  |
| R squared | 0,784 |  |  |  |  |  |  |  |  |
|  |  |  |  |  |  |  |  |  |  |
| Analysis of Variance | SS | DF | MS | F (DFn, DFd) |  |  |  |  |  |
| Regression | 60392 | 7 | 8627 | F (7, 9) = 4,666 |  |  |  |  |  |
| Residual | 16642 | 9 | 1849 |  |  |  |  |  |  |
| Total | 77034 | 16 |  |  |  |  |  |  |  |
|  |  |  |  |  |  |  |  |  |  |
| Multicollinearity | Variable | VIF | R2 with other variables |  | P value |  |  |  |  |
| β0 | Intercept |  |  |  | P=0,0181 |  |  |  |  |
| β1 | B : IL-4 | 1,306 | 0,2346 |  |  |  |  |  |  |
| β2 | C : IL-17 | 1,588 | 0,3703 |  |  |  |  |  |  |
| β3 | D : IL-25 | 1,224 | 0,1829 |  |  |  |  |  |  |
| β4 | E : IL-33 | 1,328 | 0,2471 |  |  |  |  |  |  |
| β5 | F : TSLP | 1,512 | 0,3386 |  |  |  |  |  |  |
| β6 | G : FeNO | 1,271 | 0,2131 |  |  |  |  |  |  |
| β7 | H : IgE | 1,247 | 0,1978 |  |  |  |  |  |  |
| Correlation matrix | Variable | β0 | β1 | β2 |  |  |  |  |  |
| β0 | Intercept | 1 |  |  |  |  |  |  |  |
| β1 | B : IL-4 | -0,4032 | 1 |  |  |  |  |  |  |
| β2 | C : IL-17 | -0,7381 | 0,03878 | 1 |  |  |  |  |  |
| β3 | D : IL-25 | -0,3983 | 0,3123 | 0,00588 |  |  |  |  |  |
| β4 | E : IL-33 | -0,1234 | -0,1976 | -0,06709 | β3 | β4 | β5 | β6 | β7 |
| β5 | F : TSLP | -0,7138 | 0,1866 | 0,3303 |  |  |  |  |  |
| β6 | G : FeNO | -0,01558 | -0,01894 | -0,3537 |  |  |  |  |  |
| β7 | H : IgE | -0,2962 | 0,1306 | 0,3487 |  |  |  |  |  |
| Normality of Residuals | Statistics | P value | Passed normality test (α=0,05)? | P value summary | 0,1364 | 1 |  |  |  |
| Anderson-Darling (A2*) | 0,3306 | 0,4783 | Yes | ns | 0,1064 | 0,3082 | 1 |  |  |
| D'Agostino-Pearson omnibus (K2) | 2,035 | 0,3615 | Yes | ns | 0,1578 | 0,0837 | 0,0990 | 1 |  |
| Shapiro-Wilk (W) | 0,9383 | 0,2984 | Yes | ns | 0,111 | -0,1993 | -0,0507 | -0,204 | 1 |
| Kolmogorov-Smirnov (distance) | 0,1289 | >0,1000 | Yes | ns |  |  |  |  |  |
| Data summary |  |  |  |  |  |  |  |  |  |
| Rows in table | 75 |  |  |  |  |  |  |  |  |
| Rows skipped (missing data) | 58 |  |  |  |  |  |  |  |  |
| Rows analyzed (# cases) | 17 |  |  |  |  |  |  |  |  |
| Number of parameter estimates | 8 |  |  |  |  |  |  |  |  |
| #cases/#parameters | 2,1 |  |  |  |  |  |  |  |  |

**Table S1.** Multiple linear regression coefficients, standard errors, and statistical significance for the association between Hsp70 protein level (dependent variable) and immunological markers (IL-4, IL-17, IL-25, IL-33, TSLP, FeNO, IgE) in bronchial asthma patients
